# Do Cancer-Related Fatigue and Physical Activity Vary by Age for Black Women With a History of Breast Cancer?

**DOI:** 10.5888/pcd14.170128

**Published:** 2017-11-30

**Authors:** Melody Swen, Amandeep Mann, Raheem J. Paxton, Lorraine T. Dean

**Affiliations:** 1 Department of Epidemiology, Johns Hopkins Bloomberg School of Public Health, Baltimore, Maryland; 2 Department of Biostatistics, Drexel University Dornsife School of Public Health, Philadelphia, Pennsylvania; 3 Department of Community Medicine and Population Health, University of Alabama, Tuscaloosa, Alabama; 4 Department of Oncology, Johns Hopkins School of Medicine and Sidney Kimmel Cancer Center, Baltimore, Maryland

## Abstract

**Introduction:**

Cancer-related fatigue (CRF) is the most uncomfortable symptom among women with a history of breast cancer. Black women are more likely than women of other racial/ethnic groups to have CRF risk factors, such as physical inactivity and obesity, yet CRF studies have not focused on black women. We conducted a cross-sectional analysis to assess CRF and physical activity among black women survivors of breast cancer.

**Method:**

In May and July of 2012, 267 members (mean age, 54 y) of the Sisters Network, Inc, completed an online survey of sociodemographic characteristics, medical characteristics, and physical activity, and a fatigue instrument (the Functional Assessment of Chronic Illness Therapy [FACIT]). Multiple linear regression assessed fatigue and physical activity compliance (ie, 150 minutes of moderate to vigorous physical activity per week).

**Results:**

Participants had an average FACIT score of 32.3, Fatigue was greater (*P* < .001) among the 56% of women not meeting physical activity guidelines. In multivariable analysis, correlates of fatigue showed that physical activity compliance (β = 3.20, *P* < .001) and older age group (50–59 y: β = 3.98, *P* = .001; ≥60 y,: β = 3.76, *P* = .003) were associated with less fatigue. The interaction between age and fatigue was also significant: mean differences in fatigue by physical activity level were obvious only among women younger than 50 years. (*P* < .001).

**Conclusion:**

Physical activity compliance was associated with a lower level of fatigue. However, the effect of physical activity on fatigue may differ by age. Interventions aimed at curbing CRF in black women should consider age-appropriate strategies that can be integrated into existing lifestyles.

## Introduction

Cancer-related fatigue (CRF) is considered the most uncomfortable symptom experienced by women with a history of breast cancer, a population that in 2015 exceeded 3.1 million in the United States (1,2). Compared with fatigue experienced by women without a cancer history, CRF is chronic and is not relieved by rest. Approximately 25% of breast cancer survivors experience CRF that persists for 10 years or more after an initial breast cancer diagnosis (3–5). CRF disrupts work, sleep, and social relationships, contributing to deficits in quality of life (1,3).

Correlates of CRF are a high body mass index (BMI), adjuvant radiation therapy, time elapsed since treatment completion, breast cancer recurrence, and comorbid conditions such as diabetes and cardiovascular disease (3,6–8). CRF prevalence may be higher in younger women than older women with a history of cancer (9–14). Physical activity is a mitigating factor for CRF (7,15–17).

Disparities in CRF may exist. Black women in particular may experience greater levels of CRF than women of other racial/ethnic groups because of several factors. Black women may undergo aggressive treatment regimens needed to treat late-onset cancers (black women are more likely than women of other races to be diagnosed with late-stage breast cancer) and difficult-to-treat cancers (eg, estrogen-receptor-negative or triple negative tumors) (18). In addition, black women are more likely than women of other racial/ethnic groups to be inactive (ie, to engage in <150 min/wk of moderate-intensity physical activity), to be overweight before starting treatment, and to gain more weight during treatment (19–22). These factors may place black women at increased risk for aggressive treatments, which can exacerbate CRF (22). Few studies have examined factors that may protect black women against CRF.

The objective of this study was to examine the relationship between physical activity and CRF in black women with a history of breast cancer. We hypothesized that women engaging in recommended physical activity levels would report lower levels of fatigue than women who were less active. We also hoped to determine factors that put black women at risk for CRF.

## Methods

### Sample

The study sample was drawn from the Sisters Network Inc, the largest black/African-American breast cancer survivorship organization in the United States. Participants were recruited in May, June, and July 2012 via multiple emails and by posting of anonymous links to our survey on social media blog sites affiliated with the Sisters Network. The potential reach of the email messages was 16,000 members in the Sisters Network database, which includes approximately 3,800 breast cancer survivors and about 12,100 black women without a history of breast cancer. Links posted on Facebook, the Sisters Network social network site, and Twitter were sent to approximately 6,800 women. A total of 525 of a possible 3,800 breast cancer survivors responded to our web-based study. All surveys for this cross-sectional study were completed by using Survey Monkey, a web-based platform that allows investigators to create surveys, perform routine updates, and manage survey responses. Inclusion criteria were 1) receiving a diagnosis of invasive operable breast cancer, 2) being aged 18 to 80 years at the time of the survey, 3) receiving a diagnosis of stage I to stage III C breast cancer, and 4) consenting to the web-based survey administration. Details on identification and recruitment of these women were published previously (20,21). Institutional review board approval was obtained from the University of Texas MD Anderson Cancer Center before data collection. We also obtained approval to analyze these data from the University of Alabama and the University of North Texas Health Science Center institutional review boards. All participants were treated in compliance with ethical standards. Informed consent was obtained from all participants.

### Measures

**Cancer-related fatigue (CRF).** The fatigue outcome variable was reported as a score on the Functional Assessment of Chronic Illness Therapy (FACIT) fatigue scale (Version 4). The FACIT fatigue scale is a validated 13-item self-report measure of the level of fatigue experienced during usual daily activities over the past 7 days. The scale consists of statements on level of fatigue, such as “I feel fatigue,” “I feel weak all over,” and “I feel tired,” rated on a Likert-type response scale (0 = very much fatigued to 4 = not at all fatigued). Positively worded items were reverse scored. The score was calculated by summing the individual item scores for each participant, multiplying by 13, and dividing by the number of questions answered. Higher scores indicated less fatigue, with a score range of 4 to 52. The mean score for a similar age-matched population of women in the United States is 40 (23). We used the fatigue scale to accurately capture fatigue characteristics of black women in the United States with a history of breast cancer.

**Physical activity compliance.** Physical activity compliance was assessed via a self-administered survey instrument designed for the Women’s Health Initiative (20,23). The instrument consists of 9 items that assesses recreational walking and light, moderate, and vigorous physical activity by measuring frequency and duration of physical activity. Estimates of metabolic equivalents (METs) for physical activity were calculated separately for light (METs <3.0), moderate (METs = 3.0–5.9), and vigorous (METs ≥6.0) activities. A variable was also created for moderate to vigorous physical activity (METs ≥3.0), which was then used to create a dichotomous variable (meeting or not meeting physical activity guidelines) based on a cutoff of 10.0 MET hours per week, which equaled approximately 150 minutes per week of moderate-paced walking or the equivalent of other physical activity durations and intensities. The cutoff used in this study was consistent with the current guidelines of the Centers for Disease Control and Prevention for physical activity (24). We opted to use evidence-based guidelines rather than continuous physical activity because the guidelines offer a standard that could be used to compare those meeting a clinically meaningful threshold of physical activity, thus, allowing our results to be compared with other studies of physical activity and CRF.

**Demographic and treatment factors.** Participants self-reported their age in years, height in inches, and weight in pounds. Employment status was reclassified as “working for pay” if the participant reported working outside the home even if they were retired or “not working for pay” if the participant was unemployed or was retired. Age was reclassified into 3 roughly proportional groups (<50 y, 50–59 y, and ≥60 y). Additionally, study participants self-reported the following treatment-related factors: age at diagnosis (in years), number of years since treatment completion, cancer stage (I–IIIC), cancer recurrence, and type of primary and adjuvant cancer treatments received (surgery, chemotherapy, radiation therapy, or hormone therapy). Age at the time of study and years since treatment were only moderately correlated (*r* = 0.44), so both were retained. Participants reported other chronic conditions (comorbidities), including diabetes, high cholesterol, osteoporosis, high blood pressure, and arthritis. Number of comorbidities were tabulated on the basis of the count of comorbid conditions that participants indicated. Participants who self-reported never smoking or having quit smoking were classified as “current non-smoker.” Income was separated into 7 categories: less than $20,000, $20,000 to $34,999, $35000 to $49,999, $50,000 to $64,999, $65,000 to $79,999, $80,000 to $99,999, and $100,000 or more. Age at cancer diagnosis was coded as number of years since breast cancer diagnosis, and education was coded as high school diploma or any college degree (bachelor’s degree or higher).

### Statistical analysis

Data were cleaned and analyzed using Stata version 13 (StataCorp LLC). We examined sociodemographic, lifestyle, and cancer diagnosis and treatment characteristics by physical activity compliance level by using *t* tests, χ^2^ tests, and analysis of variance tests. With continuous FACIT score as the outcome, stepwise selection determined the final set of variables in the linear regression analysis. Variables with more than 5% of missing data on income, age at cancer diagnosis, and education were excluded from analysis. We included an interaction term to test for moderation by age category. Then we generated an interaction plot to explore the relationship between physical activity and fatigue by age categories. Significance was set at *P* < .05 with a 2-sided test.

## Results

Of 3,800 possible participants, 525 initiated the study, 307 completed the survey, and 267 had sufficient data on the variables used in this analysis. The mean FACIT score was 32.3, (Table 1). Participants in the sample were on average aged 54 years, had a mean BMI of 30.4 kg/m^2^, which is considered obese, and had 1 comorbidity. Most participants worked for pay, did not smoke, were diagnosed with breast cancer at Stage II or higher, and had undergone adjuvant chemotherapy or adjuvant radiation; just under half received hormone therapy. The mean time since treatment completion was 7 years, and 16% had a recurrence. Compared with those not meeting physical activity guidelines (n = 150), participants meeting physical activity guidelines (n = 117) were significantly more likely to have higher FACIT scores (*P* > .001), to be younger (*P* = .02); to have lower BMI (*P* = .007); to have fewer comorbidities (*P* = .009); and had more time since completion of cancer treatment (*P* = .04).

**Table 1:** Participant (N = 267) Characteristics, Study of Cancer-Related Fatigue and Physical Activity Among Black Female Breast Cancer Survivors in the United States, Overall and by Not Meeting or Meeting Physical Activity Guidelines^a^, 2012

Characteristic	Overall Sample (N = 267)	Not Meeting Guidelines (n = 150)	Meeting Guidelines (n = 117)	*P* Value^b^
**FACIT fatigue score^c^, mean (SD)**	32.3 (0.4)	30.7 (0.6)	34.2 (0.5)	<.001
**Age, y**				
<50	93 (35.0)	47 (31.0)	46 (39.0)	.02
50–59	90 (34.0)	50 (33.0)	40 (34.0)	
≥60	84 (31.0)	53 (35.0)	31 (26.0)	
**Body mass index (kg/m^2^), mean (SD)**	30.4 (0.4)	31.3 (0.5)	29.3 (0.5)	.007
**Number of comorbidities, mean (SD)**	1.3 (1.1)	1.5 (1.1)	1.1 (1.1)	.009
**Working for pay^d^**	179 (67)	96 (64.0)	83 (70.9)	.23
**Current smoker**	11 (4.1)	10 (6.7)	1 (0.9)	.10
**Cancer stage at diagnosis**				
I	87 (32.6)	49 (32.7)	38 (32.5)	.77
II	131 (49.1)	75 (50.0)	56 (47.9)	
III or IV	49 (18.4)	26 (17.3)	23 (19.7)	
**Treatment type**				
Chemotherapy	186 (69.7)	105 (70.0)	82 (70.1)	.90
Radiation	181 (67.8)	104 (69.3)	77 (65.8)	.54
Hormone therapy	130 (48.7)	76 (50.7)	54 (46.2)	.47
**Years since treatment**	7 (1)	6 (1)	7 (1.0)	.04
**Recurrence**	43 (16.1)	29 (19.3)	14 (12.0)	.11

Abbreviations: FACIT, Functional Assessment of Chronic Illness Therapy; SD, standard deviation.

a Physical activities guidelines are from Centers for Disease Control and Prevention, current as of 2008 (24). Values are n (%) unless otherwise indicated. Percentages may not sum to 100 because of rounding.

b χ^2^ test was used to determine *P* values to test the difference between women who met physical activity guidelines and those who did not.

c Lower FACIT scores indicate more fatigue; scores below 30 indicate severe fatigue. The mean FACIT score in a similar age-matched population of US women without cancer is 40 (23).

d Employed, or retired and working for pay.

The multiple linear regression model suggested that FACIT scores were 3.2 points higher for those meeting physical activity guidelines than for those not meeting guidelines (*P* < .001). Additionally, FACIT scores were about 4 points higher each for those aged 50 to 59 years (*P* < .001) and 60 years or older (*P* = .03) than for those younger than 50 years (Table 2). Another demographic factor significantly associated with FACIT score was employment status. Participants currently working for pay had on average a 2-point higher FACIT score than those not working for pay (*P* = .03). No other factors were significantly associated with CRF. However, without age in the model, more years since treatment was significantly associated with less fatigue, suggesting that age at time of study accounted for some of the variance associated with years since treatment and FACIT scores.

**Table 2:** Multiple Linear Regression of Fatigue Based on FACIT (Functional Assessment of Chronic Illness Therapy) Fatigue Score^a^, Participants (N = 267) in Study of Cancer-Related Fatigue and Physical Activity Among Black Female Breast Cancer Survivors in the United States, YEAR

Variable	β (SE) [95% CI]	*P* Value **b**
**Physical activity^c^**	3.20 (0.79) [1.62 to 4.77]	<.001
**Age, y^d^**		
50–59 (n = 90)	3.98 (0.99) [2.02 to 5.95]	<.001
≥60 (n = 84)	3.76 (1.23) [1.33 to 6.19]	.003
**BMI (kg/m^2^)**	−0.074 (0.07) [−0.21 to 0.06]	.29
**Number of comorbidities**	−0.73 (0.40) [−1.52 to 0.06]	.07
**Working for pay^e^**	2.05 (0.91) [0.24 to 3.84]	.03
**Current smoker**	−0.39 (0.73) [−1.82 to 1.05]	.59
**Stage^f^**		
Stage II	0.37 (0.91) [−1.43 to 2.17]	.68
Stage III and IV	0.44 (1.21) (−1.94 to 2.82]	.71
**Treatment**		
Chemotherapy	−1.27 (0.94) [−3.12 to 0.57]	.18
Radiation	−1.41 (0.87) [−3.13 to 0.31]	.11
Hormone treatment	0.31 (0.77) [−1.21 to 1.83]	.69
**Years since treatment**	0.12 (0.08) [−0.03 to 0.27]	.12
**Recurrence**	−1.26 (1.12) [−3.46 to 0.94[	.26

a Lower FACIT scores indicate more fatigue.

b χ^2^ test was used to determine *P* values to test the null hypothesis that the variable in question is not associated with a higher fatigue score.

c Reference is 1.0, not meeting physical activity guidelines of the Centers for Disease Control and Prevention (24).

d Reference is <50 y (n = 93).

e Employed, or retired and working for pay. Reference is unemployed/retired (1.0).

f Reference is stage I.

Age had a significant interaction with adherence to physical activity guidelines (*P* < .001). The relationship between FACIT score and physical activity compliance varied by age group (Figure). For participants aged under 50 years, those who met physical activity guidelines had higher FACIT scores. Among women aged 50 years or older, the relationship between physical activity and fatigue was not significant. Overlapping confidence intervals of predictive margins for each age category showed no difference between the upper 2 age categories (50–59 y: confidence interval [CI], 2.02–5.95; ≥60 y: CI, 1.33–6.19).

**Figure 1:**
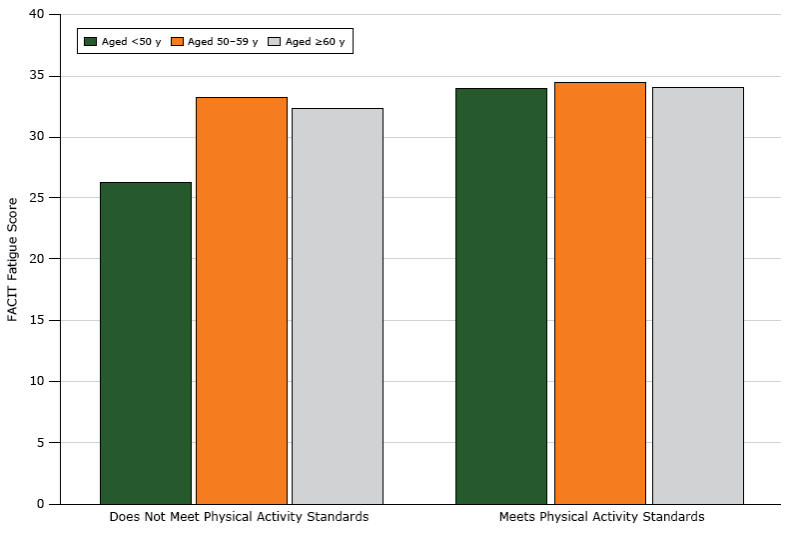
Relationship between FACIT (Functional Assessment of Chronic Illness Therapy) fatigue scores and physical activity, by age, among black female breast cancer survivors in the United States. Higher scores indicate less fatigue, with a score range of 4 to 52. The mean score for a similar age-matched population of women in the United States is 40 (23). Physical activity was assessed by a dichotomous (yes/no) variable: does not meet physical activity guidelines/meets physical activity guidelines. **Table:** 

Age, Years	Mean FACIT Score, Women Not Meeting Physical Activity Standards	Mean FACIT Score, Women Meeting Physical Activity Standards
<50	26.3	34.0
50–59	33.2	34.5
≥60	32.4	34.1

## Discussion

Meeting physical activity guidelines was associated with less CRF in this analysis of black women with a history of breast cancer. However, this association was most robust for women aged less than 50 years. As in previous studies (9–14), we found that older women had less CRF than younger women, and we also found no difference in fatigue score by physical activity level for women over 50. Study results strengthen the evidence of an association between fatigue and physical activity by age group and validate this observation for black women. Results further suggest that CRF may have more to do with a patient’s age than with how long ago the patient underwent treatment. Although more information is needed to clarify relationships for women older than 50 years, our study results can inform the development of physical activity interventions designed to remedy the consequences of CRF for black women with a history of breast cancer.

The association between fatigue and physical activity was strongest among women aged under 50 years, which may reflect that younger women (ie, diagnosed before age 45 y) may have a poorer prognosis because of greater likelihood of fast-growing, high grade, and hormone-receptor–negative tumors (25), thereby requiring more aggressive treatment. (Although we did not have data on actual age at cancer diagnosis, women aged under 50 years completing a survey for cancer survivors can be assumed to have had cancer at an early age, because having a previous cancer diagnosis was a prerequisite for study participation.) Having more aggressive treatment may increase recovery time, which compromises regular physical activity habits (24) and could result in greater CRF. Furthermore, in black women breast cancer at a younger age is often accompanied by comorbid conditions, high-dose chemotherapy, physical inactivity, and abdominal obesity. The combination of these factors may contribute to persistent mild to severe fatigue. CRF may remain elevated as a result of residual effects on cardiac function from the aggressive treatment that is often required with early onset breast cancer, which often has a stronger effect on the younger woman’s body and organ systems than on women aged 50 years or older (26).

Another possible explanation is that women under age 50 have unique demands on their time and resources that women over 50 do not and may have higher levels of CRF than older cancer survivors because of greater personal demands (ie, family and work) or greater (unrealistic) expectations for energy (9–14). More lifestyle stresses, such as demanding jobs, child care, or elder care may contribute to higher levels of fatigue (27. Younger women cite high social and environmental demands, such as working and caring for children, as a barrier to physical activity, and these demands are known to contribute to fatigue (9,28). These stresses may be exacerbated when a woman is unemployed, which was a significant factor in our sample. A possible reason is that fatigue itself may lead to an inability to work or that stress associated with lack of ability to work may contribute to fatigue. Thus, women who do engage in physical activity may be balancing existing stressors. Interventions that include educational and time-management strategies may provide women with skills to integrate physical activity into existing tasks and may serve to reduce CRF.

Randomized trials have indicated that engaging in physical activity is consistently associated with lowering CRF (7,18–21). Young female breast cancer survivors who engage in physical activity may be doing so to lower their levels of CRF. However, not all dimensions of fatigue can be remedied by physical activity alone. Fatigue may manifest itself differently in younger and older women. The impact of fatigue on learning and memory may be more relevant for younger women, because they are still actively engaged in the work force. Older women may not be bothered by or are aware of memory challenges and are more concerned with fatigue’s physical consequences (9). Younger women may also be at higher risk for depressive symptoms, insomnia, anxiety, and fear of recurrence than older breast cancer survivors (14). Thus, our finding of no difference in the relationship between physical activity levels and fatigue for women aged over 50 years may indicate that physical activity is not as effective a treatment for the kinds of fatigue that women of this age experience. As a whole, this could suggest that emotional challenges may drive higher fatigue levels for younger women with a history of cancer. Physical activity alone may not remedy that emotional fatigue. Because black women have a greater likelihood of experiencing adverse social conditions, the compounding of physical and emotional fatigue may be especially problematic over time for those experiencing cumulative stress (29). Further research is warranted to address the co-occurring roles of stress, fatigue, and depression in black women with a history of breast cancer.

Our study has limitations. As a cross-sectional study, this analysis does not establish a causal relationship between fatigue and physical activity or differentiate between the domains of physical fatigue and emotional fatigue. For example, fatigue may cause people to be less active, or physical activity may be used to decrease fatigue. To broaden the understanding of the directionality of the associations, a longitudinal study design could be used. Nevertheless, ours is a first step in identifying the relationship between fatigue and physical activity for black women. Another limitation is that we used self-reported data on fatigue, cancer history, and physical activity. Self-reported data may be subject to recall bias; however, the recall period for fatigue in the last 7 days is relatively short, and self-report of breast cancer treatment factors was validated as over 90% accurate (30). All respondents took the survey during the summer months of May, June, and July; thus, no seasonal variation in physical activity was expected to be observed. Future prospective studies could use objective measures of fatigue and physical activity. Although several FACIT scales are designed for breast cancer survivors, they focus on symptoms that are present during active cancer treatment. For this reason, those FACIT scales may not have been appropriate for this analysis of women who are further out from treatment completion. Thus, we used a general FACIT scale, which also allowed us to examine how participants compare with a similar age-matched population of women in the United States without cancer.

Our cross-sectional study explored the relationship between fatigue and physical activity by age group among black women with a history of breast cancer. Although the mean level of fatigue was slightly greater in our sample than for a similar age-matched population of women in the United States without cancer, women under 50 who met physical activity guidelines had lower levels of fatigue than those who did not meet physical activity guidelines. These results offer a platform to further examine the relationship of physical activity for black women younger than 50 years by using prospective and objective measures of fatigue and physical activity. Given our results, black women with a history of breast cancer may benefit from CRF interventions that offer physical activity options that can be integrated into existing tasks and that provide age-appropriate resources to address physical and emotional fatigue.
